# Neuronal T–type calcium channels: What's new? Iftinca: T–type channel regulation

**Published:** 2011-05-25

**Authors:** MC Iftinca

**Affiliations:** Foothills Medical Centre, Department of Pathology, CalgaryCanada

**Keywords:** calcium channel, T–type, sleep, pain, epilepsy

## Abstract

This review summarizes recent advances in our understanding of neuronal T–type calcium channel regulation as well as their physiological and pathophysiological roles. Through their ability to conduct calcium across the cellular membrane at potentials close to the resting potential, T–type calcium channels are critically important for regulating neuronal excitability, both in the central and peripheral nervous system.  T–type channels are also linked to an increasing number of neurological disorders such as the absence epilepsy and neuropathic pain.  Although there is substantial literature dealing with regulation of native T–type channels, the underlying molecular mechanism has only recently been addressed.  It is, therefore, critical to understand the cellular mechanisms that control T–type channel activity and expression, because this could provide important insight into designing novel therapeutic strategies targeting these channels.

## Introduction

Calcium is a ubiquitous intracellular second messenger critical for cellular functioning [1].  Voltage–gated calcium channels are essential mediators of rapid influx of extracellular calcium ions into the cytosol of the electrically excitable cells.  The ensuing rise in free intracellular calcium levels triggers various responses, which include the activation of calcium dependent enzymes, the secretion of neurotransmitters and hormones, as well as proliferation, differentiation, apoptosis and cell death [1, 2].  Higher organisms express multiple calcium channel subtypes in various tissues [3–27] (Table 1 and Table 2).

**Figure 1:**
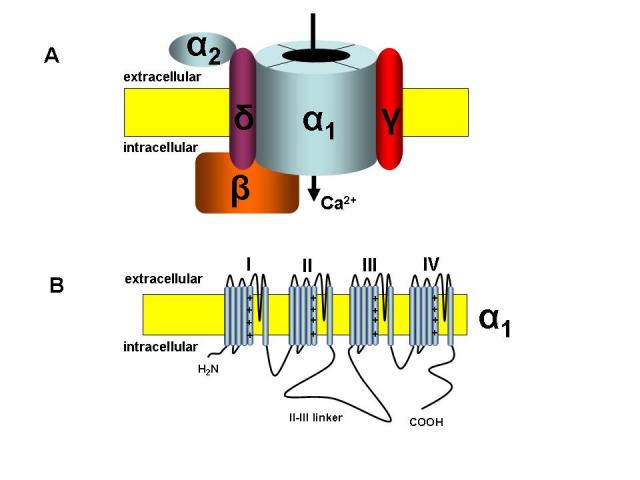
General structure of the voltage–dependent calcium channels. A. These channels are heteromultimers comprised of a pore forming alpha 1 subunit plus ancillary beta, and alpha 2–delta subunits, which co–assemble with the alpha 1 subunit to form a functional calcium channel protein. B. Transmembrane topology of the T–type calcium channel alpha 1 subunit.

The L–type calcium channels comprise the subgroup most studied, or high voltage–activated (HVA) calcium channels, which are well known, because they represent the main target of the antihypertensive drugs labelled ‘calcium channels blockers’ [28].  Other members of the HVA channel family include the N–, P–, Q– and R–types. These channel subtypes are heteromultimers comprised of a pore forming alpha 1 subunit that defines the calcium channel subtype, plus ancillary beta, and alpha 2–delta subunits which co–assemble with the alpha 1 subunit to form a functional calcium channel protein [28] (Figure 1).  All calcium channel alpha 1 subunits are made of four homologous domains (I through IV), each of which containing 6 transmembrane spanning helices (termed S1–through S6) plus a pore forming loop, that permits the selective passage of calcium ions (Figure 1).  The S4 segment in each domain contains positively charge amino acid residues in every third position (see ‘+’ signs in Figure 1) and forms the voltage sensor, which allows the channel to open and close in response to membrane potential changes.  The major transmembrane domains are linked by intra–cytoplasmatic loops.

The N– and C–termini are also localized at the cytoplasmic sides.  In contrast, T–type calcium channels, or low voltage–activated (LVA) calcium channels, represented an electrophysiological curiosity at the time when they were first described because they differ from the members of the HVA family in three key areas.  First, they require much smaller membrane depolarization close to the resting membrane potential in most excitable cells for opening [2].  Second, their unitary conductance is typically smaller [2].

Finally, unlike HVA channels, it is thought that T–type calcium channels are alpha 1 subunit monomers [2].  Also in contrast with the L–type, these channels present rapid gating kinetics and resistance to standard calcium channel blockers.

**Table 1:** Voltage–dependent calcium channel family. The family, subfamily, old nomenclature, new nomenclature and type are indicated.

HVA	Ca_v_1	Ca_v_1.1	alpha_1_S	L–type
		Ca_v_1.2	alpha_1_C	L–type
		Ca_v_1.3	alpha_1_D	L–type
		Ca_v_1.4	alpha_1_F	L–type
				
	Ca_v_2	Ca_v_2.1	alpha_1_A	P/Q–type
		Ca_v_2.2	alpha_1_B	N–type
		Ca_v_2.3	alpha_1_E	R–type
				
LVA	Ca_v_3	Ca_v_3.1	alpha_1_G	T–type
		Ca_v_3.2	alpha_1_H	T–type
		Ca_v_3.3	alpha_1_I	T–type

**Table 2:** Tissue distribution of T–type calcium channel isoforms

Tissue	Location	Isoform	Method	Reference
brain	ubiquitous	3.1,3.2,3.3	immunohistochemistry, in situ hybridization	3, 4, 5
peripheral nervous tissue	DRG, autonomic ganglia, retina, olfaction	3.1,3.2, 3.3	electrophysiology	6, 7, 8, 9
heart	myocytes (atrial, ventricular, septal) pacemaker cells (Purkinje cells, sinoatrial node cells, latent pacemaker cells)	3.1, 3.2	immunohistochemistry, mRNA, electrophysiology	10, 11, 12, 13, 14, 15
smooth muscle	vascular	3.1, 3.2	immunohistochemistry, mRNA, electrophysiology	16
	non–vascular: urinary tract (bladder, urethra, prostate), kidney (afferent and efferent arterioles, vasa recta, distal tubules, cortical collecting ducts), gastro–intestinal tract (stomach, small intestine, colon, spleen, liver), myometrium (uterus), male genitary tract (vas deferens), airway	3.1, 3.2	immunohistochemistry, mRNA, electrophysiology	17
skeletal muscle	embryonic, newborn	3.2	mRNA, electrophysiology	18,19
endothelium		3.1		20
bone	osteoblasts	3.1		21
endocrine	adrenal, pituitary, pancreas, thyroid, ovary	3.1, 3.2	immunohistochemistry, mRNA, electrophysiology	22, 23, 24, 25
sperm		3.1, 3.2	immunohistochemistry, mRNA, electrophysiology	26
lung		3.1, 3.2	mRNA, electrophysiology	27

Although these channels have been identified more than 25 years ago [6, 9, 10, 29, 30] it was not until later when the three cDNAs have been cloned, that the T–type calcium channels began to be in the centre of attention.  In vertebrates, the T–type calcium channel family encompasses three different alpha 1 subunit genes: CACNA1G, CACNA1H and CACAN1I, which encode alpha1G,alpha1H, and alpha1I, respectively (or as per current nomenclature, Cav3.1, Cav3.2 and Cav3.3, respectively) [11, 27, 31–33] with unique functional and pharmacological profiles, and specific cellular and subcellular distributions [2–5, 12, 17, 34] (Table 1 and Table 2).   Also, the analysis of the cDNAs clearly indicate that many splice variants exist for the three Cav3.1, Cav3.2 and Cav3.3 subunits which enhances the diversity of T–type channels isoforms and there is growing evidence for significant differences in the electrophysiological properties of these splice variants of a given Cav subunit [35–39].   

This review focuses on the roles and regulation of neuronal T–type channels. Our goal, however, is not to present in detail all the roles and regulations of the T–current studied so far but to highlight some of the recent advances in our understating of T–type channel regulation and their functional roles in health and disease in the nervous system. 

### Physiological and pathophysiological roles of T–type channels

**Figure 2:**
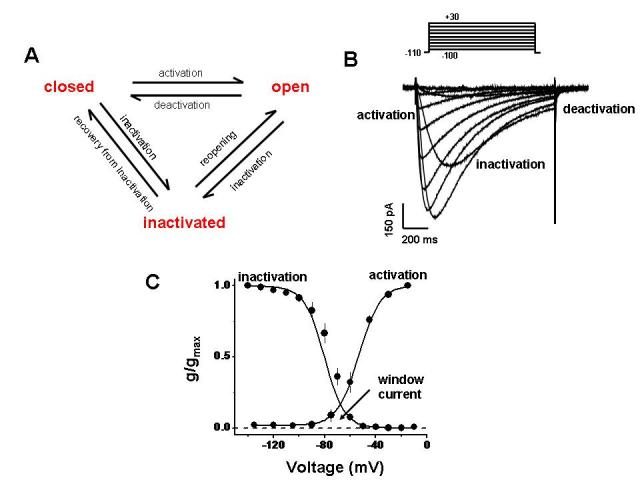
Key functional properties of T–type calcium channels. A. Simplified scheme of T–type channel gating. B. Typical family of whole cell T–type calcium currents, elicited by stepping from a holding potential of –110 mV to a series of incremental depolarizations. C. Voltage dependence of activation and inactivation.   The overlap of the activation and inactivation curves gives rise to a window current (i.e., a voltage range where T-type channels can be tonically active).

Neuronal T–type calcium channels were first identified in Purkinje and inferior olivary neurons and later in many other central neurons and in peripheral sensory neurons [6, 9, 10, 29, 30].  The role of T–type channels in regulating the neuronal firing patterns (low–threshold calcium spikes, action potential burst firing, post–hyperpolarisation rebound action potentials and burst firing) is determined by the very interesting biophysical characteristics of these channels [reviewed in 40].  Because T–type calcium channels are available for opening only from very negative membrane potentials they are ideally suited for regulating neuronal excitability [reviewed in 40 and 41] (Figure 2).

Small membrane depolarisations can trigger the opening of T–type calcium channels and calcium entry, which in turn serves to further depolarise the plasma membrane, thus initiating action potentials bursts.  The T–type currents are transient because the channels inactivate rapidly upon depolarization (Figure 2).  However, when the membrane repolarises, T–type calcium channels deactivate slowly and this permits significant calcium influx following an action potential. Paradoxically, transient membrane hyperpolarisation can also augment T–type calcium channel mediated burst activity in a phenomenon termed ‘rebound bursting’. At normal neuronal resting membrane potentials, a large portion of T–type calcium channels is tonically inactivated [2, 7] (Figure 2).  Transient membrane hyperpolarizations (such as those arising from inhibitory postsynaptic potentials, or activation of certain types of potassium channels) results in recovery of these channels from inactivation, thus increasing the number of channels that are available for opening upon subsequent membrane depolarisations and enhancing their abilities to trigger action potential bursts.  

Another interesting feature of T–type channels is the pronounced overlap in the voltage ranges of activation and inactivation.  Thus a small fraction of the channels will remain tonically active at membrane potentials within this region of overlap (which is close to the resting membrane potential) leading to the generation of the so–called window current [42] (Figure 2).  This feature has been linked to the well–documented role of T–type calcium channels in neuronal activity [42].  The intrinsic window current provides a means for cardiac automaticity, although the precise mechanism by which these channels regulate the excitability of cardiac tissue remains a subject of debate [reviewed in 13], and also provides a means for T–type channel mediated calcium entry that triggers hormone secretion in the heart, kidney and neuroendocrine tissues [22, 23, 43].

The well documented role of T–type calcium channels in regulating neuronal firing patterns occurs under normal physiological conditions, such as during sleep rhythms [44–47].  However, there is a growing body of evidence related to the roles of the T–type calcium channels under pathophysiological conditions such as epilepsy, autism, hypertension, atrial fibrillation, congenital heart failure, pain, psychoses and cancer [12, 14–17, 20, 41, 43, 48–65].  It is well established that T–type calcium channels are critical players in the development of generalized seizures in humans and animals [55, 65].  Knockout of Cav3.1 calcium channels protects mice from absence seizures [48, 57], and number of mutations have been described in Cav3.2 channels in patients with childhood absence and other forms of idiopathic generalized epilepsies [52, 55, 65]. However, when introduced into recombinant channels that are functionally expressed in heterologous systems, the functional consequences of the majority of the known Cav3.2 epilepsy mutations are subtle, and in some cases not detectable [52, 54, 56, 64]. 

Increased T–type channel activity has also been linked to neuropathic and inflammatory pain states [60].  Inhibitors of T–type calcium channels mediate analgesia when injected intrathecally into rats. Selective in vivo antisense knockdown of Cav3.2 channel antagonizes neuropathic and inflammatory pain [49, 51] whereas knockdown of the Cav3.2 and Cav3.3 reverses tactile allodynia and thermal hyperalgesia in rats [50].  Conversely, T–type calcium channel activity is upregulated during diabetic neuropathy [53]. This is consistent with T–type calcium channels playing a major role in regulating the excitability of primary afferent pain fibers. 

Recently, there has been new evidence that T–type calcium channels may be involved in other pathophysiological processes. Splawski et al. [58] described several missense mutations within CACNA1H gene linked to autism spectrum disorder.  These mutations were responsible for significantly altering the Cav3.2 channel activity by causing a positive shift in the activation properties and a reduction in conductance.  Interestingly a third of the autism spectrum disorder patients suffer from epilepsy [58]. Although autism is a complex developmental disorder it is tempting to speculate based on the role of T–type calcium channels in the neuronal development and excitability [66] that mutations in the CACNA1H gene may affect the neuronal function during neurogenesis [67] and could play a critical role in the development of autism. Another recent study explored the effects of a novel selective T–type calcium channel inhibitor, which produced antipsychotic–like activity [61]. This compound blocked the psychostimulant effects of amphetamine and MK–801 by decreasing the amphetamine–induced c–fos expression as well as MKZ–801–induced glutamate levels in the nucleus accumbens.  Basal, amphetamine– and MK–801–induced dopamine efflux was altered. These findings suggest that T–type calcium channel selective blockers could represent a novel treatment for schizophrenia. T–type calcium channel antagonists could be a viable treatment for essential tremor as demonstrated by Handforth and colleagues [62] in two mouse models.  These findings suggest a role for T–type calcium channels, especially for Cav3.1, which is the dominant subtype expressed in the inferior olive, in essential tremor.  Not only the T–type calcium channels may play a role in essential tremor but also they appear as viable targets in the treatment of parkinsonian tremor [63].

It is clear that T–type calcium channels display unique functional properties that support a number of critical cellular functions in a variety of tissues, and their dysfunction is linked to neurological consequences.  Furthermore, different cellular mechanisms are in place to fine tune T–type channel activity.   It is clear that T–type channels constitute important targets for clinically active drugs [43, 68–71].  

### Physiological regulation of T–type channel function

**Figure 3:**
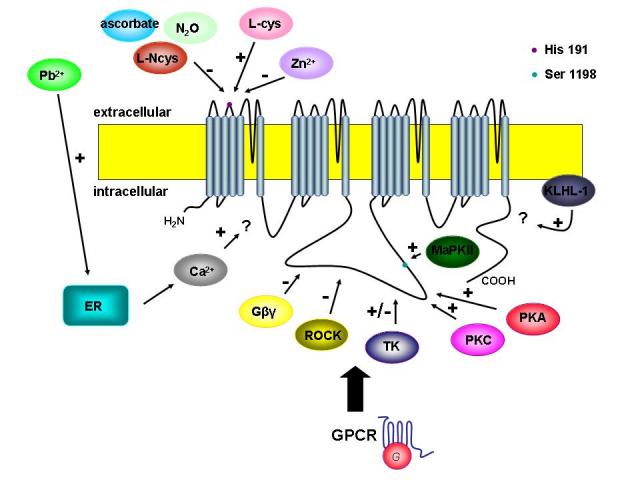
Regulation of T–type channel activity by activation of G protein–coupled receptors and subsequently second messengers, redox factors, cations and novel modulators.  Note that the final coupling mechanism between these G beta gamma subunits and the T–type channel is not understood.  Plus and minus symbols indicate the stimulation and inhibition of T–type channel activity, respectively. (GPCR – G protein–coupled receptors; PKG– protein kinase G, PKA – protein kinase A, PKC – protein kinase C, TK – tyrosine kinase, MAPKII – mitogen–associated protein kinase II, L–cys – L–cysteine; L–Ncys – L–nitrosocysteine; KLHL–1 – neuronal actin–binding protein Kelch–like 1)

There is an extensive body of evidence regarding the regulation of T–type currents in response to activation of a range of G protein coupled receptors, such as angiotensin, GABAB, muscarinic (M1), corticotropin releasing factor receptor 1 (CRFR1) and serotonin receptors which in turn activate a wide range of intracellular second messenger pathways [8, 72–79] (Figure 3).  

A number of serine/threonine kinases, calmodulin–dependent protein kinases and tyrosine kinases have been found to be involved in the regulation of T–type calcium channel.  One of the first to be described was CamKII [78], which was reported to have a selective potentiation effect on recombinant Cav3.2 currents.  The authors showed that this effect is due to a hyperpolarizing shift in the activation curve of the channel, which in turn increases the size of the window current and makes the channel more available for opening. The domain II–III linker region of the channel was the key structural motif involved in CamKII regulation (Figure 3), and subsequently Serine 1198 was identified as the key CamKII phosphorylation site involved in this effect [79].  It was also shown that Cav3.2 inhibition (but not other T–type calcium channel subtypes) by direct interactions of G protein  beta 2 and gamma subunits (G beta2 gamma) with the domain II–III linker region (Figure 3) of the channel [80] and reduces the open probability of the channel [81].  

Protein kinase C activation reportedly enhances the activities of Cav3.1, Cav3.2 and Cav3.3 calcium channels [82] with the magnitude of upregulation being dependent on Cav3 channel subtype. The PKC effect could be localized also to the domain II–III linker region of the channel (Figure 3), but the exact location of the PKC site within this region remains undefined.  Given that the CamKII effect occurred selectively for Cav3.2 and did not involve a change in the channel's activation behaviour, it is likely that the PKC acts at a distinct site from that phosphorylated by CamKII.  Activation of protein kinase A (PKA) also upregulates Cav3.2 channel activity in Xenopus oocytes (Cav3.1 and Cav3.3 were not examined in this study [83] and the domain II–III linker region was again the critical determinant of this effect.  However, the authors could not identify the key PKA site involved in the PKA–mediated upregulation.  Interestingly, in mammalian cells, both PKA and PKC were reported to potently enhance all Cav3 channel subtype, but curiously, only at near physiological temperatures, whereas no modulation could be observed at room temperature [84].  It is unclear why PKC and PKA are able to enhance T–type channel activity at room temperature in oocytes and not in mammalian cells. It is important to note that T–type channel function is highly dependent on temperature [85], which may also affect their overall susceptibility to kinase regulation.

Application of lysophospatidic acid (LPA) through activation of Rho kinase mediates a reversible inhibition of transiently expressed Cav3.1 and Cav3.3 calcium channels and a shift towards more depolarized potentials of the activation and inactivation curves for the Cav3.2 channels  [86]. The site of action of Rho kinase could be localized to two clusters of serines and threonines within the domain II–III linker region of Cav3.1 (Fig. 3) that is highly conserved across all T–type calcium channel isoforms [86].  It has been shown that LPA can induce a reduction of rebound burst activity in lateral habenula neurons that rely on Cav3.1 channels for burst activity.  Cav3.1 channels are widely distributed in the central nervous system and especially in the thalamus and neocortex [5] two of the areas known to be important for seizure genesis.  Recently it has been shown that ROCK inhibitors mediated a reduction of seizures in mice [87] pointing to a possible role for the Rho/Rho–kinase signalling pathway in epilepsy. Along these lines, LPA receptor activation has been linked to neuropathic pain [88], and it remains to be determined if this is due to altered Cav3.2 channel function

Overall, it is remarkable that a number of different second messengers act on T–type calcium channels via the domain II–III linker region. This suggests that this region is a key element involved in the regulation of T–type channel function. This also may provide a means by which clinically relevant T–type calcium channel mutations in this region could mediate neurophysiological effects without directly affecting the biophysical properties of the channels.  

T–type calcium channels can also be regulated by pathways that do not involve classical intracellular messengers (Figure 3). Reducing agents such as the endogenous amino acid L–cysteine have been shown to upregulate T–type currents in nociceptive neurons, thus triggering the development of hyperalgesia [88] due to an increase in excitability of these neurons [90].   This type of redox modulation was shown to occur selectively for Cav3.2 channels, and could also be demonstrated in reticular thalamic neurons [90]. Conversely, oxidizing agents inhibited Cav3.2 calcium channels [91]. Moreover, T–type calcium channel inhibition and inhibition of burst firing of reticular thalamic was also observed upon extracellular, application of a series of endogenous s–nitrosothiol reagents  such s L–nitrosocysteine [92], and by oxidizing agents such as ascorbate [93].  In the case of ascorbate, the mechanism of action was shown to involve an oxidization of a unique histidine residue (His191) in the domain I S3–S4 region of the Cav3.2 channel (Figure 3).   Interestingly, the same histidine residue was shown to be involved in the augmentation of Cav3.2 currents in response to L–cysteine but also in inhibition of the of the Cav3.2 currents in response to nitrous oxide (N2O). L–cysteine was shown to chelate endogenous zinc ions that normally inhibit Cav3.2 channel activity thus relieving this inhibition and increasing current activity [94]. Nitrous oxide on the other hand is believed to be involved in the production of reactive oxygen species, which in turn are likely to oxidize H191 of CaV3.2 in a localized metal–catalyzed oxidation reaction [95]. It should also be noted that in addition to the potent zinc mediated inhibition of Cav3.2 channels (IC50 aprox 800 nM), zinc ions cause an aprox 100 fold slowing of Cav3.3 tail currents which culminates in increased Cav3.3 channel during action potential bursts [96]. These observations are particularly interesting when considering recent findings showing that the chelation of zinc ions can alter the occurrence and frequency of epileptiform discharges [97]. The authors suggested that this was the result of zinc–mediated modification of the gating kinetics of the Cav3.3 channel, a T–type channel isoform, highly expressed in the thalamic neurons [5].

Recently, the lead ion was shown to have an excitatory effect on T–type calcium channel activity and thereby on action potential firing of pyramidal neurons in CA1 region of the rat hippocampal slices [98]. This effect was very much different from that of zinc in that it appeared to involve the release of calcium from the internal stores through the inositol trisphosphate and ryanodine receptors.

Endogenous modulators derived from amino acids appear to be key regulators of T–type channel function with actions that are inextricably linked to zinc inhibition of these channels.  The findings obtained with redox and oxidizing agents also serve to further underscore the role of Cav3.2 T–type channel activity in pathophysiological states such as pain and epilepsy. 

Hildebrandt et al. have recently shown that activation of M1 muscarinic acetylcholine receptors selectively inhibits Cav3.3 channel activity [74]. The authors showed that this inhibition was mediated by G alpha q /G alpha 11 linked pathways and partially involved G beta gamma subunits. The M1 mediated modulation did not involve any of the major second messenger pathways, such as PKC, PKA, tyrosine kinases, PI3 kinases or calmodulin dependent protein kinase, or PIP2. Alltogether, this suggests a novel pathway for T–type channel regulation that needs to be explored further.  One possibility is a redundant inhibitory mechanism that in which two separate pathways are activated by M1 receptors concomitantly and each being capable of inhibiting channel activity.   Such a mechanism would be consistent with the authors' observation that multiple channel regions appeared to be involved [74]. 

Another recent study described a regulation of Cav3.2 channels by corticotrophin releasing factor (CRF) receptors [76].  The authors showed that activation of co–expressed CRF1 receptors in HEK293T cells selectively inhibited Cav3.2 calcium channels. The inhibition was dependent on G beta gamma subunits activated by a cholera toxin sensitive G alpha pathway. However, the observed modulation differed from the aforementioned inhibition mediated by G beta 2 gamma in that a leftward shift in the half inactivation potential was observed.  Such a shift in the steady–state inactivation curve will lead to a decrease in size of the window current and therefore a reduced T–type channel availability for opening. This suggests that the CRF1 receptor mediated regulation of Cav3.2 channels is unique. Agonists of the CRF receptors have been implicated in regulating sleep rythms [99]. Considering the well established role of the T–type channel in thalamic oscillations it is tempting to speculate that the observed effect of the CRF receptor activation on T–type channel gating may play a role in the regulation of sleep rhythms.  A recent report by Kim and colleagues [100] showed that CRF receptor activation of T–type channels expressed in MN9D cells (a cell line with characteristics of dopaminergic neurons) results in inhibition of T–type channels that is dependent on PKC activity. This suggests that not only may the coupling between CRF receptors and T–type channel differ depending on the cellular environment, but also that effects of PKC activation may be highly dependent on cell type (recall the PKC mediated enhancement of T–type channels described above). It is possible that CRF receptor activation will produce different results depending on the T–type channel splice isoform.

Aromoralaran et al. [101] that the neuronal actin-binding protein (ABP) Kelch–like 1 (KLHL1) selectively increases Cav3.2 current density and deactivation kinetics. These changes lead to an overall increase in the calcium influx, through these channels, without a change in conductance or open probability. KLHL1 is a constitutive protein, which is widespread in the brain and contributes to the modulation of pacemaker activities, short burst firing, and low–threshold calcium spikes [67].  KLHL1 also participates in neurite outgrowth and its genetic elimination in Purkinje neurons leads to dendritic atrophy and motor insufficiency. This is another novel regulatory mechanism and due to the large distribution of KLHL1 and T–type channel in the brain, it would be interesting to assess its contribution in pathophysiological instances. 

In spite of the many new insights into the T–type channel regulation gained in the last years our current understanding of the precise underlying molecular mechanisms remains relatively limited, and the putative vast majority of the pathways regulating native channels have not yet been reconstituted in heterologous expression systems.  

### Regulation of T–type channel expression

The subcellular distribution of T–type calcium channels across the central nervous system varies with T–type calcium channel subtype and brain region [3, 5]. For example, in neocortical pyramidal cells, Cav3.1 channels show a mainly somatic distribution, whereas Cav3.3 channels are expressed at both the soma, as well as the proximal and distal dendritic trees [3].  The molecular mechanisms that underlie the differential subcellular distribution of individual calcium channel subtypes are at this point unknown, as are the cellular mechanisms that regulate the trafficking of T–type calcium channels to the plasma membrane.  Co–expression of Cav3.1 calcium channel alpha 1 subunit with HVA calcium channel beta and alpha 2–delta subunits increases alpha 1 subunit surface expression levels [102, 103] and recently Bae and colleagues [104] showed a weak association between Cav3.3 calcium channel alpha 1 subunit and the beta subunit. However, no physical association among these subunits has ever been demonstrated in vivo.  It is very possible that the different types of T–type channel are able to associate with different interacting proteins, which in turn may well affect the extent of membrane trafficking, and the specific targeting to appropriate subcellular loci.  

It has been shown that increased Cav3.2 channel membrane expression occurs for channels carrying a number of different childhood absence epilepsy mutations [105].  It is unclear, however, whether this is due to altered ER retention, or due to increased membrane stability. There is also evidence for an increase in the T–type channel expression, in CA1 pyramidal neurons in a rat model of temporal lobe epilepsy [106]. Direct effects of these epilepsy mutations on membrane expression or effects on interactions with regulatory proteins that are involved in channel targeting could potentially account for their pathophysiological impact even in the absence of any alterations in channel biophysics. Another mechanism for a change in surface expression of T–type calcium channels could be mediated by the various hormonal changes that occur in epilepsy patients [107]. It has been shown that 17 beta–estradiol treatment induces an increase in the mRNA expression, which leads to increased functional expression of Cav3.1 and increased burst firing in hypothalamic neurons in a guinea pig model [108]. It is possible that such a mechanism could underlie the increased T–type calcium channel expression that is observed in mouse models of absence epilepsy [57]. While potentially contributing to pathophysiology, altered regulating of either the extent of trafficking of the channels to the plasma membrane, or their stability in the plasma membrane may perhaps provide novel means of modulating T–type calcium channel activity for therapeutic purposes.

An upregulation in T–type calcium channel expression also appears in painful diabetic neuropathy in dorsal DRG sensory neurons [53]. Conversely, knockdown of Cav3.2 and Cav3.3 localized to the spinal cord in rats mediates potent analgesia [49, 50].  Therefore, it is tempting to speculate that blocking the trafficking of these channels to the cellular membrane could decrease or even completely abolish neuropathic pain.

Channel expression can be regulated by alternate splicing events [36–39]. Alternate splice isoforms of each Cav3 channel subtype have been reported, and were shown to give rise to channels with distinct functional properties [36–39]. The distribution of specific splice variants across the brain and other tissues, and whether any such expression patterns may vary, the developmental state remaining yet to be described. A recent study has provided intriguing evidence that a number of childhood absence epilepsy mutations may affect the mRNA splicing of the Cav3.2 calcium channel gene, by eliminating splice junctions [38]. This in turn may give rise to inappropriate splice variants in specific tissues, which may then result in altered neuronal function [38]. Along these lines, when conducting mutagenesis studies, it is essential that the mutations are introduced into the appropriate splice variant, as effects of such mutations may manifest only in certain channel variants.

Overall, although our understanding of the molecular mechanisms that regulate T–type channel expression at to mRNA and protein levels remain poorly understood. Alterations in the normal T–type channel expression patterns may well play significant roles in T–type channel pathophysiology and a clear possibility emerges that T–type calcium channels can be good targets for controlling pain and absence seizures by using a genetic approach.   

## Concluding remarks

T–type calcium channels are critical contributors to membrane excitability in both neuronal and non-neuronal cells, and conversely, aberrant T–type calcium channel function has been linked to a number of different disorders.  Although there is now an increasing understanding of the molecular determinants that underlie regulation of T–type channels by a range of second messenger pathways, the intricate mechanisms that control, T–type channel expression and distribution remain largely unknown. Today with the help of Cav3.1 and Cav3.2 knock out animals, we will be able to further explore the involvement of T–type calcium channels in physiological and pathophysiological states. The Cav3.1 knock out mouse already helped to further our understanding of the major role of T–type calcium channels in sleep and absence epilepsy by regulating the neuronal burst firing in the thalamocortical relay neurons [44, 45, 48] while the Cav3.2–/– mice confirmed the role of the Cav3.2 in nociception [51]. Unfortunately, no report has been published yet to show the functional consequences of Cav3.3 knock–out.

The effects of the changes in T–type channel activity could also be related to changes in potassium channel activity. Small– and large–conductance calcium–activated potassium channels (SKCa and BKCa) have been shown to be functionally coupled in neurons and vascular smooth muscle cells were they are involved in regulating neuronal firing patterns and vasodilation, respectively [109–112]. Recently, voltage–activated potassium channels (Kv) have also been found to be coupled not only functionally but also physically to the T–type calcium channels [113, 114].  

The search for selective or subtype–specific blockers for T–type channels has been going on for years.  However, these compounds are not yet available.  Many T–type channel blockers have been described in the treatment of neurological and cardiovascular disorders.  These compounds belong to various drug classes (dihydropyridines, succinimide derivatives, diphenylbutylpiperidine derivatives, bendodiazepines, anesthetics) but their action is not selective enough for the T–type channel [68–71]. The search for new generations of selective T–type channel blockers is ongoing [43, 70, 71]. Due to the fact that T–type calcium channels have isofor–specific properties, the need to design drugs selective for a given T–channel subtype has emerged. The challenge will then be to determine whether specific T–channel isoforms/variants can be selectively targeted for therapeutic intervention. Although a new route has been started recently, with the utilization of genetic approaches, such as gene knockout by gene targeting or gene knockdown by antisense techniques [44, 48–50, 57], it does not represent a viable therapeutic approach at this time.  Understanding the regulatory mechanisms of the T–type calcium channel regulation and expression may lead to the identification of novel means of regulating T–type channel activity for therapeutic purposes, especially for controlling pain and absence seizures, thus underscoring the importance of closing these gaps in our current knowledge.
